# Regional disparities in lymphedema treatment and access to complex decongestive therapy: a nationwide survey in Japan

**DOI:** 10.1093/jjco/hyaf120

**Published:** 2025-07-24

**Authors:** Mariko Masujima, Shinsuke Akita, Makiko Tazaki, Akane Tsujimoto, Ryoko Katagiri, Chikao Yasuda, Tetsuya Tsuji

**Affiliations:** Graduate School of Nursing, Chiba University, 1-8-1, Inohana, Chuo-ku, Chiba-shi, Chiba 260-8672, Japan; Department of Plastic, Reconstructive, and Aesthetic Surgery, Graduate School of Medicine, 1-8-1, Inohana, Chuo-ku, Chiba-shi, Chiba University, Chiba 260-8670, Japan; Graduate School of Nursing, Chiba University, 1-8-1, Inohana, Chuo-ku, Chiba-shi, Chiba 260-8672, Japan; Graduate School of Nursing, Chiba University, 1-8-1, Inohana, Chuo-ku, Chiba-shi, Chiba 260-8672, Japan; Faculty of Informatics, Graduate School of Informatics, Chiba University, 1-33, Yayoicho, Inage-ku, Chiba-shi, Chiba 263-8522, Japan; Department of Vascular Surgery, Japan Community Healthcare Organization Hoshigaoka Medical Center, 4-8-1, Hoshigaoka, Hirakata-shi, Osaka 573-8511, Japan; Department of Rehabilitation Medicine, Keio University School of Medicine, 35, Shinanomachi, Shinjuku-ku, Tokyo 160-8582, Japan

**Keywords:** lymphedema treatment, questionnaire survey, complex decongestive therapy, regional disparity

## Abstract

**Background:**

Lymphedema has a significant impact on patient quality of life. However, it remains unclear whether the provision of lymphedema treatment in Japan is uniform across regions. This study aimed to clarify the current situation regarding lymphedema treatment with emphasis on complex decongestive therapies (CDT) availability and implementation in Japan.

**Methods:**

A nationwide web-based survey was conducted. Respondents included healthcare professionals from designated cancer care hospitals and other medical institutions treating lymphedema in Japan. The distribution of variables, including the implementation of lymphedema treatment, was compared between designated cancer care hospitals and other facilities using the chi-square test. Japan was divided into nine regions to compare and analyze access to medical institutions providing CDT for lymphedema on both inpatient and outpatient bases.

**Results:**

Of the 372 facility responses analyzed, ˃95% reported treating secondary lymphedema of the extremities, whereas ˂30% treated head and neck lymphedema. The number of CDT inpatients per 100 000 people in the region with the lowest patient volume was approximately 2% of that in the region with the highest volume. Similarly, the number of CDT outpatients per 100 000 people in the lowest-volume region was one-third of that in the highest-volume region. There was no significant correlation between facilities with high outpatient numbers and those with low outpatient numbers (ρ =0.57, *P*-value = 0.11).

**Conclusion:**

Eliminating regional disparities in access to lymphedema treatment facilities, particularly for inpatient CDT, would improve quality of life and enable patients to manage the condition regardless of where they live.

## Teaser

A nationwide survey revealed the current state of lymphedema treatment and regional characteristics of facilities implementing complex decongestive therapy for lymphedema.

## Introduction

Lymphedema, a chronic condition characterized by tissue swelling due to lymphatic system impairment, can be caused by cancer or cancer treatment and can have a profound impact on the quality of life of cancer survivors [1–3]. In Japan, one million people are diagnosed with cancer each year [4]. Breast cancer, the leading cause of upper limb lymphedema, has the highest incidence rate among all cancers in women. The incidence rates of gynecological and prostate cancers—the leading causes of lower limb lymphedema—are also high; furthermore, it is estimated that hundreds of thousands of cancer survivors suffer from lymphedema [5–7].

Early detection and treatment of lymphedema are crucial. The Ministry of Health, Labor and Welfare has established designated cancer care hospitals to ensure that patients receive high-quality cancer treatment and medical care to improve their quality of life, regardless of where they live in Japan [8]. As lymphedema significantly impacts patient quality of life, these designated cancer care hospitals indicate whether they provide specialized lymphedema outpatient services. The first stage of lymphedema treatment is complex decongestive therapy (CDT), which involves four elements: skin care, compression therapy using bandages and compression underwear, manual lymph drainage, and exercise [9]. CDT has been shown to improve quality of life for patients with lymphedema [10,11].

Since 2016, national health insurance in Japan has covered conservative lymphedema treatment when provided by healthcare professionals with specialized CDT training. Previous studies found that less than half of designated cancer care hospitals had lymphedema outpatient departments [12,13]. In recent years, the importance of lymphedema treatment has become widely recognized in society, and the number of medical facilities and clinics that diagnose and treat lymphedema has increased, even outside of designated cancer care hospitals. However, no comprehensive nationwide study has provided an overview of the distribution of medical institutions offering lymphedema treatment.

Therefore, in this study, we conducted a comprehensive survey of lymphedema treatment facilities throughout Japan to address the lack of nationwide data on regional disparities in lymphedema treatment, including access to CDT. Clarifying the uneven distribution of treatment facilities for CDT by the type of medical institution is essential for formulating strategies to prevent the progression of lymphedema and enable patients to lead healthy lives.

## Materials and methods

### Study design and participants

We implemented a comprehensive descriptive quantitative cross-sectional study to examine lymphedema treatment, as it is affected by the functions and roles of various medical institutions, doctors from various departments, and professionals who carry out CDT. A nationwide web-based survey was conducted among 456 designated cancer care hospitals and other medical institutions in Japan to ensure a broad representation of current lymphedema treatment practices.

Participant recruitment was conducted by mail to designated cancer care hospitals and by email to the members of four Japanese academic societies specializing in lymphedema: the Japan Society of Plastic and Reconstructive Surgery, the Japanese Lymphedema Society, the Japanese Society for Lymphedema Therapy, and the Japanese Society of Lymphology. An opt-out system was used for research implementation as the research did not involve personal patient information. Each facility designated a medical professional with comprehensive knowledge of lymphedema treatment at their institution as the respondent, and they completed the survey via Google Forms. The data collection period was from November 2023 to March 2024.

### Variables

The questionnaire was developed by a multidisciplinary research team comprising doctors, nurses, physiotherapists, and occupational therapists who collectively specialized in lymphedema management. To evaluate the validity of the questionnaire, a pilot test was conducted at three medical institutions at the forefront of lymphedema treatment, and the questionnaire was subsequently finalized. This methodological approach ensured that the questionnaire was comprehensive and inclusive, covering 34 questions across four domains: facility overview, CDT for Lymphedema, surgical treatment for lymphedema, and open-ended questions about lymphedema treatment. In questions regarding the number of patients with lymphedema, respondents were asked to provide the actual number of patients rather than the cumulative number of outpatients and inpatients.

Institutional demographic data collected included the name and address of each facility to divide the area. This information was used to eliminate potential duplicates among responses and to identify the geographical region within Japan. Plastic surgeons analyzed the results of the questionnaire sections on surgical treatment, while nursing researchers analyzed the results of the questionnaire components focused on CDT for lymphedema. The distribution of responding facilities by treatment modality—specifically those using only conservative treatment, those using only surgical treatment, and those implementing both conservative and surgical treatments—was considered essential data for the study; hence, these metrics were included. This study was approved by the Ethics Committee of Chiba University School of Medicine (Ethics Review Number M10663).

### Statistical analysis

The distribution of variables, including the availability of lymphedema treatment, facility location, treatment settings, consultations from other facilities, insurance coverage, department of lymphedema management, treatable areas or subjects, and available treatments, are outlined in Supplemental Table S1. These distributions were compared between designated cancer care hospitals and other facilities using a chi-square test, with *P* < 0.05 considered statistically significant. Fisher’s exact test was applied for contingency tables containing cells with a value below 5.

For regional analysis, we also compared and analyzed the access to medical institutions providing CDT for lymphedema across geographic regions throughout Japan. The country was divided into nine regions: Hokkaido, Tohoku, Kanto, Hokuriku, Chubu, Kinki, Chugoku, Shikoku, and Kyushu (including Okinawa Prefecture). We compared the number of medical institutions that provide CDT to inpatients and the total number of inpatients with lymphedema per year by region. Additionally, we assessed the number of medical facilities providing CDT on an outpatient basis and the total number of outpatients with lymphedema per 100 000 people per year across each region.

Furthermore, data on the number of annual outpatients who received CDT did not follow the normal distribution, thereby spearman's rank correlation was used to examine the correlation between the number of annual outpatients who received CDT between designated cancer care hospitals and other types of facilities, and between high- and low-volume facilities. The high/low-volume cut-off was set at the 75th percentile of the total number of outpatients. All statistical analyses were performed using IBM SPSS Statistics version 29 software (IBM Corp., Armonk, NY, USA).

## Results

### Survey summary

A total of 376 responses were received, of which 372 facilities agreed to participate in the survey and were included as valid responses. Out of 456 designated cancer care hospitals, 252 (response rate: 55.3%) responded, but one facility did not respond to the survey and was excluded from the analysis, leaving 251 facilities included in the analysis. Furthermore, 124 responses came from facilities affiliated with a relevant academic society. There were no overlapping facilities with designated cancer care hospitals, and after excluding three facilities that refused to participate in the survey, the analysis included 121 non-designated facilities. Of the final analysis included 372 facilities, 279 facilities (75.0%) responded providing lymphedema treatment, while 93 (25.0%) indicated they did not provide such treatment (Supplemental Table S2).

### Current states of lymphedema treatment in general

Of the 279 facilities that treated lymphedema, 201 (72%) were designated cancer care hospitals (Table 1). Regarding treatment settings, 99% of both designated cancer care hospitals and other medical institutions treated patients with lymphedema care on an outpatient basis. Approximately 60% of designated cancer care hospitals and 50% of other medical institutions offered inpatient services for lymphedema management. The rate of accepting referrals from other hospitals rate was significant higher for other medical institutions (88.5%) compared to designated cancer care hospitals (49.8%), indicating a high referral acceptance rate.

**Table 1:** Current state of lymphedema treatment.

		Total facilities (n = 279)			Designated cancer care hospital (n = 201)			Other medical institution (n = 78)			*P-*value
		n (%)			n (%)			n (%)			
Treatment setting^a^											
	Outpatient	276	(98.9)		199	(99.0)		77	(98.7)		<0.001
	Inpatient	157	(56.3)		119	(59.2)		38	(48.7)		
	Home visit nursing services	10	(3.6)		3	(1.5)		7	(9.0)		
	House call medical services	8	(2.9)		0	(0.0)		8	(10.3)		
	Other	3	(1.1)		2	(1.0)		1	(1.3)		
Referral from other facilities											
	Available	169	(60.6)		100	(49.8)		69	(88.5)		0.000
	Not available	71	(25.4)		67	(33.3)		4	(5.1)		
	Other (depends on department)	39	(14.0)		34	(16.9)		5	(6.4)		
Department of Lymphedema Management^a^											
	Plastic surgery	143	(51.3)		110	(54.7)		33	(42.3)		<0.001
	Breast	103	(36.9)		85	(42.3)		18	(23.1)		
	Gynecology	79	(28.3)		73	(36.3)		6	(7.7)		
	Vascular surgery	35	(12.5)		23	(11.4)		12	(15.4)		
	Rehabilitation	34	(12.2)		30	(14.9)		4	(5.1)		
	Palliative medicine	19	(6.8)		13	(6.5)		6	(7.7)		
	Other	52	(18.6)		32	(15.9)		20	(25.6)		
Treatable areas or subjects^a^		(n = 277)			(n = 199)			(n = 78)			
	Upper limb lymphedema (Secondary)	272	(98.2)		196	(98.5)		76	(97.4)		0.001
	Lower limb lymphedema (Secondary)	264	(95.3)		190	(95.5)		74	(94.9)		
	Upper limb lymphedema (Primary)	161	(58.1)		106	(53.3)		55	(70.5)		
	Lower limb lymphedema (Primary)	168	(60.6)		110	(55.3)		58	(74.4)		
	Genital lymphedema	135	(48.7)		88	(44.2)		47	(60.3)		
	Trunk lymphedema	98	(35.4)		64	(32.2)		34	(43.6)		
	Head and neck lymphedema	76	(27.4)		53	(26.6)		23	(29.5)		
	Pediatric primary lymphedema	60	(21.7)		38	(19.1)		22	(28.2)		
	Pediatric lymphatic malformation	37	(13.4)		25	(12.6)		12	(15.4)		
	Other	3	(1.1)		3	(1.5)		0	(0.0)		
Available treatment^a^		(n = 273)			(n = 197)			(n = 76)			
	CDT	249	(91.2)		176	(89.3)		73	(96.1)		0.65
	Surgical treatment	128	(46.9)		97	(49.2)		31	(40.8)		
	Interventional radiology (IVR)	5	(1.8)		2	(1.0)		3	(3.0)		
	Others	4	(1.5)		4	(2.0)		0	(0.0)		

CDT: Complex Decongestive Therapy.

^a^Multiple answers were allowed.

The most common department responsible for lymphedema management at both designated cancer care hospitals and other medical institutions was plastic surgery. More than 95% of all facilities reported capability to treat secondary lymphedema of the upper and lower limbs. However, ˂50% of facilities indicated ability to treat genital lymphedema and head and neck lymphedema. Treatment of primary lymphedema for the upper and lower limbs was significantly more common at other medical institutions than at designated cancer care hospitals. Approximately 90% of both designated cancer care hospitals and other medical institutions provided CDT, and approximately half provided surgical treatment, with no significant difference in treatment modalities between facility types.

**Figure 1:**
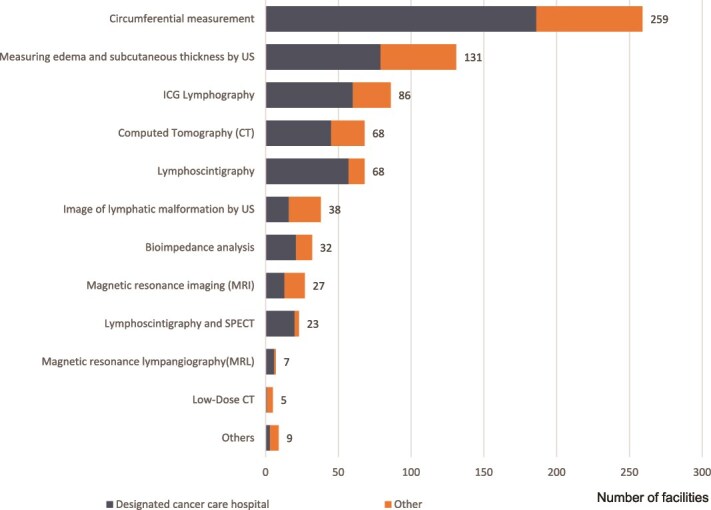
The standard diagnostic test for lymphedema. Multiple answers allowed (n = 271). US: Ultrasound; ICG: Indocyanine Green; SPECT: Single Photon Emission Computed Tomography.

Figure 1 shows the diagnose methods used for lymphedema. The most common was circumferential measurement, implemented at 259 facilities (96%), followed by ultrasound assessment of edema and subcutaneous thickness at 131 facilities (48.3%) and indocyanine green lymphography at 86 facilities (31.7%).

### Summary of CDT for lymphedema

**Figure 2:**
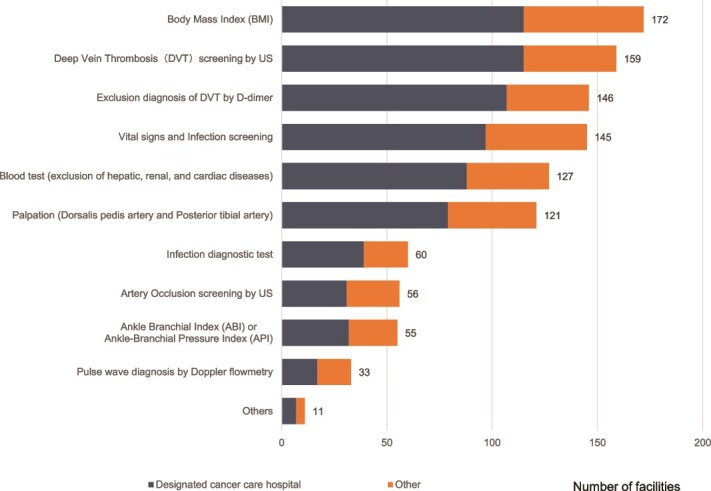
Tests or screening items carried out before CDT for lymphedema. Multiple answers allowed (n = 229). US: Ultrasound.

The most common assessment was the Body Mass Index calculation (75%). Additionally, over 60% of facilities also screened for Deep Vein Thrombosis (DVT) using ultrasound, D-dimer, vital signs, and infection markers to assess CDT suitability (Fig. 2).

Insurance coverage for CDT was available at about half of all facilities (Table 2). CDT coverage was significantly higher in designated cancer care hospitals than in other medical institutions (62.6% vs. 32.8%). the combined rates of partial coverage and non-coverage, as well as the rate of complete non-coverage, were significantly higher in other types of medical institutions than in designated cancer care hospitals.

**Table 2:** Insurance coverage for CDT.

Insurance coverage	Total (n = 230)		Designated cancer care hospital (n = 163)		Other medical institution (n = 67)		*P-*value
	n	(%)	n	(%)	n	(%)	
Covered	124	(53.9)	102	(62.6)	22	(32.8)	<0.001
Both covered and not covered	64	(27.8)	36	(22.1)	28	(41.8)	0.002
Not covered	30	(13.0)	16	(9.8)	14	(20.9)	0.023
Other	12	(5.2)	9	(5.5)	3	(4.5)	1.0

CDT: Complex Decongestive Therapy.

### Regional access to CDT for lymphedema for inpatients and outpatients

The facilities implementing CDT in Japan were divided into nine regions, as shown in Table 3. The Kanto region had the most facilities (⁓30% of the total)—six times more than Shikoku, which had the fewest cancer treatment facilities. Kanto had 12 times more facilities than Hokuriku and Shikoku combined, which had the fewest other types of medical institutions.

**Table 3:** Facilities capable of CDT in nine regions of Japan.

Region	Total (n = 249)		Designated cancer care hospital (n = 176)		Other medical institution (n = 73)	
	n	(%)	n	(%)	n	(%)
Hokkaido	16	(6.4)	12	(6.8)	4	(5.5)
Tohoku	19	(7.6)	15	(8.5)	4	(5.5)
Kanto	72	(28.9)	48	(27.3)	24	(32.9)
Hokuriku	12	(4.8)	10	(5.7)	2	(2.7)
Chubu	29	(11.6)	25	(14.2)	4	(5.5)
Kinki	40	(16.1)	20	(11.4)	20	(27.4)
Chugoku	19	(7.6)	15	(8.5)	4	(5.5)
Shikoku	10	(4.0)	8	(4.5)	2	(2.7)
Kyushu	32	(12.9)	23	(13.1)	9	(12.3)

CDT: Complex Decongestive Therapy.

**Figure 3:**
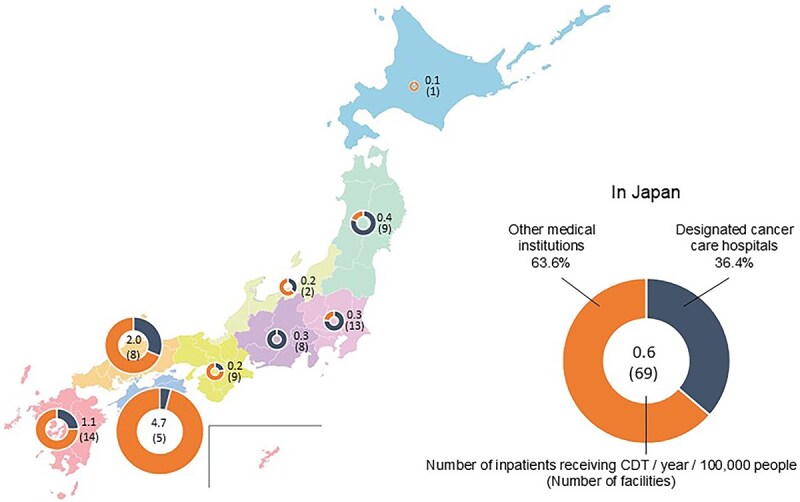
Number of inpatients receiving CDT for lymphedema per 100 000 people per year in each region. The number of hospitalized patients receiving CDT for lymphedema per 100 000 people per annum in each region of Japan was presented as a numerical value on a donut chart. The number of facilities was indicated by the number in brackets within the circle.

The number of inpatients receiving CDT for lymphedema per 100 000 people in each of Japan’s nine regions is shown in Fig. 3. Shikoku, Chugoku, and Kyusyu had the highest number of inpatients per 100 000 people. Hokkaido had the lowest number of inpatients per 100 000 (0.1/100000 population, across 1 facility), and there was an approximately 2% inpatient rate in Shikoku. A total of 726 inpatients (mean: 0, 0–164, IQR: 0–1) were admitted annually, across 69 facilities (36.4% were designated cancer care hospitals, while 63.6% were other medical institutions) that admitted inpatients and performed CDT in Supplemental Fig. S1. Shikoku, Kyushu, and Chugoku had the highest number of inpatients, while Hokkaido had the lowest, with only three patients at one facility (less than one-fiftieth of the highest regional total).

**Figure 4:**
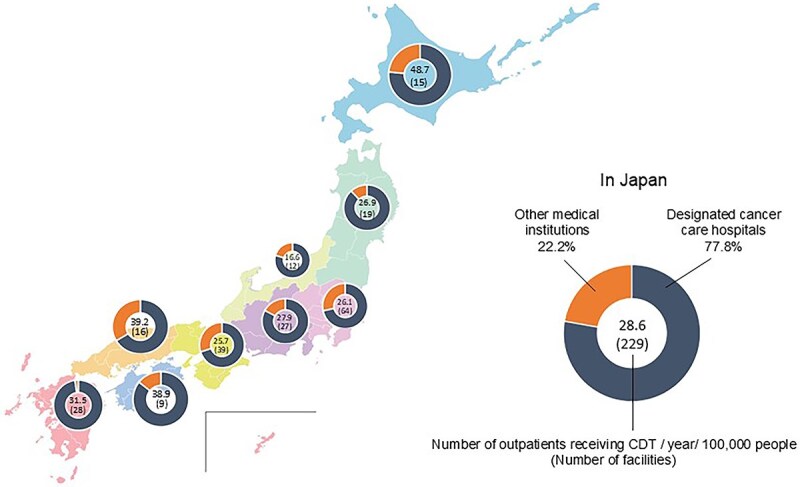
Number of outpatients receiving CDT for lymphedema per 100 000 people per year in each region. The number of outpatients receiving CDT for lymphedema per 100 000 people per annum in each region of Japan was presented as a numerical value on a donut chart. The number of facilities is indicated by the number in brackets within the circle.

The number of outpatients and facilities per 100 000 people in each of Japan’s nine regions is shown in Fig. 4. There were 35 689 outpatients per year (mean: 60, 0–1381, IQR: 20–198), treated at 229 facilities performing CDT for outpatients (77.8% were designated cancer care hospitals and 22.2% were other medical institutions). Hokkaido, Chugoku, and Shikoku had the highest number of outpatients per 100 000 people. Hokuriku had the lowest number of outpatients per 100 000 (16.6/100 000 population, across 12 facilities), approximately one-third of the outpatient rate in Hokkaido.

We also analyzed the regional distribution of outpatients receiving CDT relative to population density. Spearman’s rank correlation was used to compare the number of outpatients receiving CDT between designated cancer care hospitals and other medical institutions, as well as between high-volume centers (≥199 or more CDT procedures performed annually, representing the 75th percentile of annual CDT volumes) and low-volume centers. Results showed no significant correlation between high-volume and low-volume centers (ρ = 0.57, *P-*value = 0.11; Supplemental Table S3).

## Discussion

In this study, we investigated the current state of lymphedema treatment and CDT use through a nationwide questionnaire survey of medical facilities and designated cancer care hospitals involved in lymphedema treatment. The results suggest that, depending on the location and type of lymphedema, some treatment facilities may be unable to meet patient demand. This geographic variation is particularly evident in regional disparities that exist in CDT implementation for lymphedema among inpatients and outpatients.

Our survey results on lymphedema treatment showed that almost all medical facilities treat cancer-related lymphedema of the extremities. However, few facilities treat genital lymphedema, head and neck lymphedema, primary lymphedema, or lymphedema in children. Improved transparency from medical facilities regarding their capability to treat these specific types of lymphedemas could help patients more readily to access appropriate care.

Currently, CDT is eligible for insurance coverage; however, some facilities still provide treatment as self-paid services. Compared to the results of a previous study at designated cancer care hospitals [12] in 2017, which showed a 10% coverage rate for medical insurance, the results of our survey show a fivefold increase in coverage. However, while there have been no previous surveys of the rate of medical insurance coverage at other medical institutions, this rate remains low in non-designated medical institutions. For future research, it is necessary to investigate the reasons for the lack of CDT insurance coverage and to develop strategies to eliminate the cost disparity for patients receiving CDT.

There is a 50-fold difference in the number of inpatients with lymphedema admitted annually in Western Japan for CDT treatment between the regions with the lowest and highest values. Our results suggest that patients who require hospitalization cross regional boundaries to receive treatment when there are no appropriate facilities in their area. Intensive inpatient CDT under medical supervision is essential for severe lymphedema [14]. It is equally important to promote the use of outpatient lymphedema clinics that are easily accessible to patients to prevent lymphedema progression [15]. The present study analyzed the number of outpatient CDT patients as a proportion of the population and showed no significant differences between regions. Future interventions should focus on improving facilities for severe lymphedema patients and enhancing cooperation between medical institutions, especially in eastern Japan.

This study presents a broader picture of the current status of lymphedema treatment in Japan. We received responses from approximately half of the designated cancer care hospitals, which exceeded the number of facilities that responded in previous studies [12,13] by ˃50. Furthermore, according to the National Cancer Center [16], as of September 2023, 261 (57.0%) of the 456 designated cancer care hospitals had outpatient departments providing lymphedema treatment. This survey was answered by 201 designated cancer care hospitals that provide lymphedema treatment. This means that 77.0% of designated cancer care hospitals with lymphedema outpatient departments participated in this survey. Although these figures do not match exactly, the survey results were considered to generally reflect the current state of lymphedema treatment in designated cancer care hospitals in Japan. We also received responses from approximately 80 other medical institutions providing lymphedema treatment. While existing studies have focused on lymphedema treatment at designated cancer care hospitals, this survey more accurately reflects the current situation.

Our study has several limitations. First, the response rate for “other medical institutions” recruited via members of specific academic societies was unknown. Additionally, this questionnaire did not ask about bed count. It is presumed that the size of other medical institutions differs from that of designated cancer care hospitals. The next survey should include a question about bed count to more thoroughly examine regional disparities in inpatient numbers. In the future, establishing a registration system that comprehensively captures “other medical institutions” will enable a more accurate depiction of the current status of lymphedema treatment in Japan.

Second, the responding facilities self-reported the number of outpatients and inpatients who received CDT. If a nationwide database of patients with lymphedema were established, more accurate surveys could be conducted on the current state of lymphedema treatment.

The data obtained in this study showed that facilities capable of treating secondary lymphedema of the extremities were established nationwide, but few facilities could treat primary lymphedema. Regional disparities existed in the inpatient treatment using CDT as well as between facilities regarding the application of CDT insurance coverage. Eliminating regional disparities in inpatient lymphedema treatment facilities will improve the quality of life of patients receiving lymphedema treatment wherever they live.

## Supplementary Material

Suppl_Fig_S1_hyaf120

Suppl_Table_S1_hyaf120

Suppl_Table_S2_hyaf120

Suppl_Table_S3_hyaf120
