# Supplementary material for: Regional disparities in lymphedema treatment and access to complex decongestive therapy: a nationwide survey in Japan
Source: Jpn J Clin Oncol. 2025 Jul 24;55(11):1267–73. doi: 10.1093/jjco/hyaf120 (PMC12596720; doi:10.1093/jjco/hyaf120)
Supplement: Suppl_Fig_S1_hyaf120 [file suppl_fig_s1_hyaf120.pptx]

## Slide 1
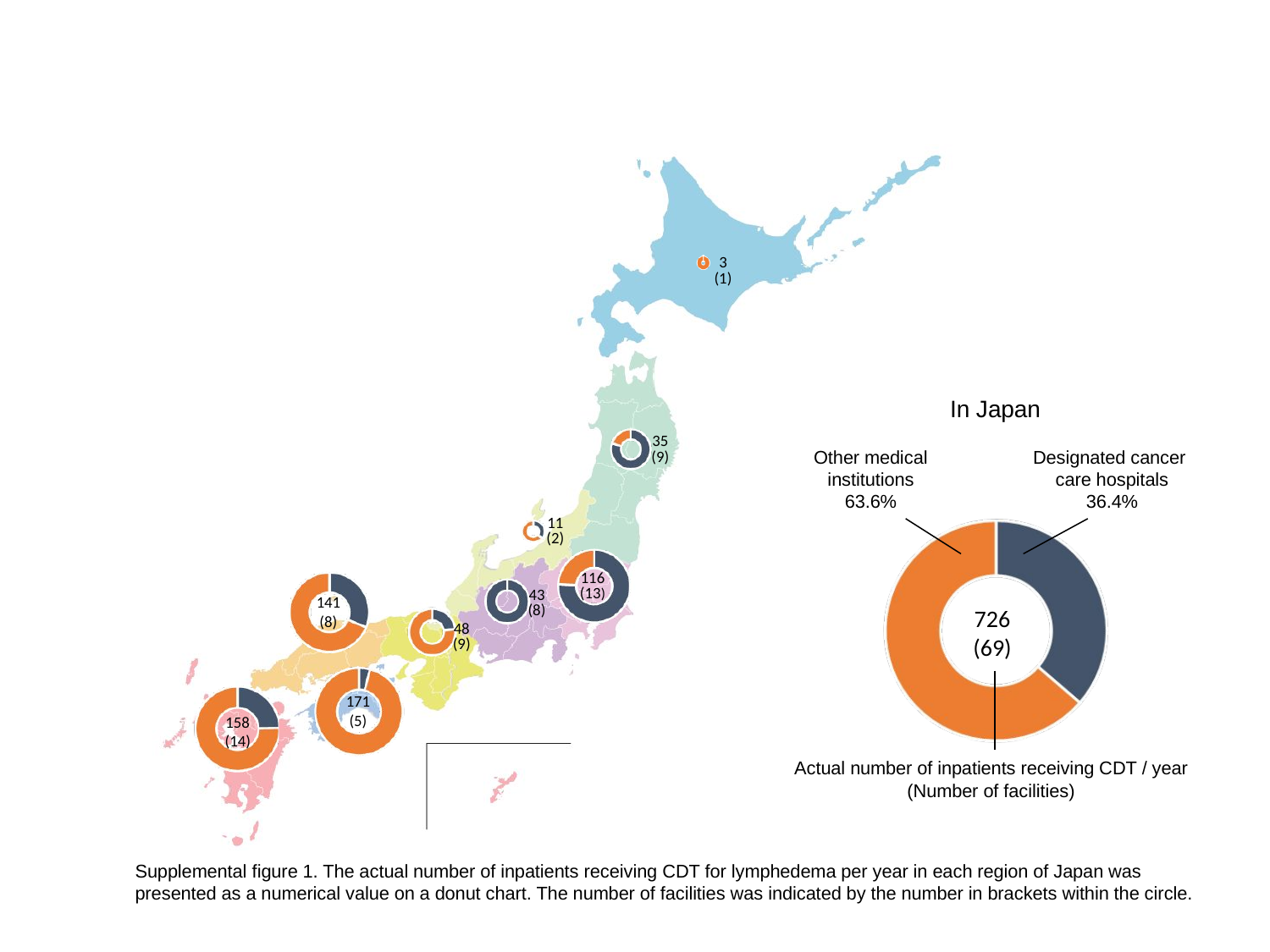

3
(1)
In Japan
35
(9)
Designated cancer
care hospitals
36.4%
Other medical institutions
63.6%
11
(2)
116
(13)
43
(8)
141
(8)
726
(69)
48
(9)
171
(5)
158
(14)
Actual number of inpatients receiving CDT / year
(Number of facilities)
Supplemental figure 1. The actual number of inpatients receiving CDT for lymphedema per year in each region of Japan was presented as a numerical value on a donut chart. The number of facilities was indicated by the number in brackets within the circle.
