# Supplementary material for: Regional disparities in lymphedema treatment and access to complex decongestive therapy: a nationwide survey in Japan
Source: Jpn J Clin Oncol. 2025 Jul 24;55(11):1267–73. doi: 10.1093/jjco/hyaf120 (PMC12596720; doi:10.1093/jjco/hyaf120)
Supplement: Suppl_Table_S1_hyaf120 [file suppl_table_s1_hyaf120.docx]

**Supplemental Table 1.**

**List of questions regarding content related to the current state of lymphedema treatment and CDT for lymphedema**

| Please answer all questions regarding the department of lymphedema management. | |
| --- | --- |
|  | Gynecology, Breast, Rehabilitation, Vascular surgery, Plastic surgery, Palliative Medicine, and Others |
| Please select the option regarding the insurance coverage for lymphedema treatment at your facility. | |
|  | Covered, Not covered, Both covered and not covered, and Others |
| Please select the options that apply to your treatment setting. | |
|  | Outpatient, Inpatient, Home visit Nursing Services, House call medical services, and Others |
| Please select the options that apply to your ability to take referrals from other facilities. | |
|  | Available, Unavailable, and Department dependent |
| Please select which standard diagnostic tests your facility does for lymphedema. | |
|  | Circumferential measurement, Bioimpedance analysis, Measuring edema and subcutaneous thickness by ultrasound, Image of lymphatic malformation by Ultrasound, Lymphoscintigraphy, Lymphoscintigraphy and Single-Photon Emission Computed Tomography, Magnetic Resonance Imaging, Magnetic Resonance Lymphangiography, Computed Tomography, Low-Dose Computed Tomography, Indocyanine Green Lymphography, and Others |
| Please select which treatments are available for lymphedema at your facility. | |
|  | Complex Decongestive Therapy, Surgical treatment, Interventional radiology (IVR), and Others |
| Please select which treatable areas or subjects apply to your facility. | |
|  | Upper limb, Lymphedema (Secondary), Lower limb lymphedema (Secondary), Upper limb lymphedema (Primary), Lower limb lymphedema (Primary), Pediatric primary Lymphedema, Pediatric lymphatic malformation, Head and neck Lymphedema, Trunk lymphedema, Genital Lymphedema, and Others |
| Please select which tests or screening items are carried out before CDT for lymphedema is undertaken. | |
|  | Body Mass Index, Ankle Branchial Index or Ankle-Branchial Pressure Index, Palpation (Dorsalis pedis artery and Posterior tibial artery), Pulse wave diagnosis by Doppler flowmetry, Blood test (exclusion of hepatic, renal, and cardiac diseases), Vital signs and Infection screening, Infection diagnostic test, Exclusion diagnosis of Deep Vein Thrombosis by D-dimer, Deep Vein Thrombosis screening by Ultrasound, Artery Occlusion screening by Ultrasound, and Others |
| Please enter the actual number of cases per year of CDT hospitalization. | |
|  | January to December 2022 |
| Please enter the actual number of cases per year of CDT outpatients. | |
|  | January to December 2022 |

CDT: Complex Decongestive Therapy
