# Supplementary material for: Regional disparities in lymphedema treatment and access to complex decongestive therapy: a nationwide survey in Japan
Source: Jpn J Clin Oncol. 2025 Jul 24;55(11):1267–73. doi: 10.1093/jjco/hyaf120 (PMC12596720; doi:10.1093/jjco/hyaf120)
Supplement: Suppl_Table_S2_hyaf120 [file suppl_table_s2_hyaf120.docx]

**Supplemental Table 2. Summary of Survey**

|  | Type of facility | Total |  | Designated cancer care hospital | | Other  medical institution | |
| --- | --- | --- | --- | --- | --- | --- | --- |
|  |  | n=372 |  | n=251 |  | n=121 |  |
|  |  | n | (%) | n | (%) | n | (%) |
| Availability of lymphedema treatment | | | |  |  |  |  |
|  | available | 279 | (75.0) | 201 | (80.1) | 78 | (64.5) |
|  | not available | 93 | (25.0) | 50 | (19.9) | 43 | (35.5) |
|  |  |  |  |  |  |  |  |
| Respondent Occupation^＊^ | | | |  |  |  |  |
|  | Doctor | 229 | (61.6) | 147 | (58.6) | 82 | (67.8) |
|  | Nurse | 95 | (25.5) | 74 | (29.5) | 21 | (17.4) |
|  | Physical Therapist | 21 | (5.6) | 12 | (4.8) | 9 | (7.4) |
|  | Occupational Therapist | 8 | (2.2) | 6 | (2.4) | 2 | (1.7) |
|  | Massage Therapist | 3 | (0.8) | 0 | (0.0) | 3 | (2.5) |
|  | Other | 22 | (5.9) | 16 | (6.4) | 6 | (5.0) |
|  |  |  |  |  |  |  |  |
| Location of facility | |  |  |  |  |  |  |
|  | Hokkaido | 18 | (4.8) | 14 | (5.6) | 4 | (3.3) |
|  | Tohoku | 29 | (7.8) | 25 | (10.0) | 4 | (3.3) |
|  | Kanto | 110 | (29.6) | 68 | (27.1) | 42 | (34.7) |
|  | Hokuriku | 18 | (4.8) | 14 | (5.6) | 4 | (3.3) |
|  | Chubu | 49 | (13.2) | 38 | (15.1) | 11 | (9.1) |
|  | Kinki | 58 | (15.6) | 29 | (11.6) | 29 | (24.0) |
|  | Chugoku | 30 | (8.1) | 22 | (8.8) | 8 | (6.6) |
|  | Shikoku | 15 | (4.0) | 11 | (4.4) | 4 | (3.3) |
|  | Kyushu and Okinawa | 45 | (12.1) | 30 | (12.0) | 15 | (12.4) |

^＊^Multiple answers were allowed.
