# Supplementary material for: Regional disparities in lymphedema treatment and access to complex decongestive therapy: a nationwide survey in Japan
Source: Jpn J Clin Oncol. 2025 Jul 24;55(11):1267–73. doi: 10.1093/jjco/hyaf120 (PMC12596720; doi:10.1093/jjco/hyaf120)
Supplement: Suppl_Table_S3_hyaf120 [file suppl_table_s3_hyaf120.docx]

**Supplemental Table 3.**

Analysis of the impact of designated cancer care hospitals and high-volume centers on the number of outpatients receiving CDT per capita across regions

| Region | CDT per one hundred thousand population | Proportion of designated cancer care hospital | The proportion of high-volume hospital  (≧199） | |
| --- | --- | --- | --- | --- |
| Hokkaido | 48.7 | 0.77 | 0.81 | |
| Tohoku | 26.9 | 0.88 | 0.78 | |
| Kanto | 26.1 | 0.72 | 0.80 | |
| Hokuriku | 16.6 | 0.80 | 0.24 | |
| Chubu | 27.9 | 0.83 | 0.61 | |
| Kinki | 25.7 | 0.70 | 0.61 | |
| Chugoku | 39.2 | 0.66 | 0.78 | |
| Shikoku | 38.9 | 0.86 | 0.75 | |
| Kyushu | 31.5 | 0.98 | 0.82 | |
| Spearman’s rank correlation ρ | | 0.03 | | 0.57 |
| *P*-value | | 0.93 | | 0.11 |
